# *Caenorhabditis elegans* Cyclin D/CDK4 and Cyclin E/CDK2 Induce Distinct Cell Cycle Re-Entry Programs in Differentiated Muscle Cells

**DOI:** 10.1371/journal.pgen.1002362

**Published:** 2011-11-10

**Authors:** Jerome Korzelius, Inge The, Suzan Ruijtenberg, Martine B. W. Prinsen, Vincent Portegijs, Teije C. Middelkoop, Marian J. Groot Koerkamp, Frank C. P. Holstege, Mike Boxem, Sander van den Heuvel

**Affiliations:** 1 Developmental Biology, Utrecht University, Utrecht, The Netherlands; 2 Molecular Cancer Research, University Medical Center Utrecht, Utrecht, The Netherlands; University of California San Diego, United States of America

## Abstract

Cell proliferation and differentiation are regulated in a highly coordinated and inverse manner during development and tissue homeostasis. Terminal differentiation usually coincides with cell cycle exit and is thought to engage stable transcriptional repression of cell cycle genes. Here, we examine the robustness of the post-mitotic state, using *Caenorhabditis elegans* muscle cells as a model. We found that expression of a G1 Cyclin and CDK initiates cell cycle re-entry in muscle cells without interfering with the differentiated state. Cyclin D/CDK4 (CYD-1/CDK-4) expression was sufficient to induce DNA synthesis in muscle cells, in contrast to Cyclin E/CDK2 (CYE-1/CDK-2), which triggered mitotic events. Tissue-specific gene-expression profiling and single molecule FISH experiments revealed that Cyclin D and E kinases activate an extensive and overlapping set of cell cycle genes in muscle, yet failed to induce some key activators of G1/S progression. Surprisingly, CYD-1/CDK-4 also induced an additional set of genes primarily associated with growth and metabolism, which were not activated by CYE-1/CDK-2. Moreover, CYD-1/CDK-4 expression also down-regulated a large number of genes enriched for catabolic functions. These results highlight distinct functions for the two G1 Cyclin/CDK complexes and reveal a previously unknown activity of Cyclin D/CDK-4 in regulating metabolic gene expression. Furthermore, our data demonstrate that many cell cycle genes can still be transcriptionally induced in post-mitotic muscle cells, while maintenance of the post-mitotic state might depend on stable repression of a limited number of critical cell cycle regulators.

## Author Summary

During development, cells face the important decision whether to continue to proliferate, or to exit the cell-division cycle and fully differentiate. Improved insight into the molecular mechanisms that arrest the cell cycle during terminal differentiation is important for our understanding of normal development, as well as for cancer research and regenerative medicine. To investigate the arrested state of terminally differentiated cells, we examined muscle cells in the model organism *C. elegans*, which is known for its reproducible cell-division pattern. We found that expression of a single cell cycle kinase with its regulatory partner (Cyclin) induced many cell division genes in muscle. While Cyclin D and E kinases often act similarly, only Cyclin D with CDK-4 triggered DNA replication in muscle, and this combination induced a much broader transcriptional response than Cyclin E/CDK-2. Despite activation of a substantial cell cycle program, Cyclin/CDK expression did not induce complete muscle cell division and failed to induce some key cell cycle regulators. Our results highlight distinct activities of Cyclin D and Cyclin E kinases, and they indicate that cell-cycle gene expression remains remarkably flexible in differentiated cells. We propose that the post-mitotic state of differentiated cells is maintained by tight control of a few regulatory genes.

## Introduction

During the terminal stages of differentiation, cells usually arrest proliferation and permanently exit the division cycle. Insight in how this post-mitotic state is established and maintained is of both fundamental and clinical importance. Entry into the cell cycle requires activation of Cyclin Dependent Kinases (CDKs) in the G1 phase of the cell cycle. These kinases promote expression of cell cycle genes that are controlled by E2F/DP (together named “E2F”) transcription factors (for review: [1]). CDK inhibitors (CKIs), such as p21^Cip1^ and p27^Kip1^, counteract cell cycle entry through association with Cyclin/CDK complexes and inhibition of their activity. In addition, members of the retinoblastoma protein (pRb) family inhibit cell cycle entry through repression of E2F-regulated cell cycle genes [2]. When activated in the G1 phase, Cyclin D/CDK4(6) and Cyclin E/CDK2 kinases phosphorylate pRb, which prevents its association with E2F and allows activating E2F transcription factors to induce S phase gene expression. Differentiation signals are thought to induce cell cycle arrest through activation of negative regulators of G1 progression, probably in parallel with chromatin remodeling and modification complexes that induce stable repression of cell cycle genes [3]–[5].

Only a few examples have been reported of post-mitotic cells that re-enter the cell cycle while maintaining the differentiated state. In *Drosophila,* expression of E2F together with the Cdc25c phosphatase String or Cyclin E/CDK2 induces continued division of differentiated cells during eye and wing development [6]. In mammals, loss of the pRb tumor suppressor allows proliferation of certain terminally differentiated cells, such as the post-mitotic hair cells of the mouse inner ear [7]. In addition, inactivation of pRb family members can result in the development of retinoblastoma or related tumors from fully differentiated neurons [8], [9]. Thus, at least some terminally differentiated cells that are normally arrested can be induced to initiate cell proliferation with no apparent de-differentiation.

In this study, we use the nematode *C. elegans* to examine the cell cycle arrest associated with terminal differentiation. *C. elegans* shows a tight inverse relationship between proliferation and differentiation, and a highly reproducible pattern of terminal differentiation [10], [11]. Except for 55 ‘blast’ cells, all cells differentiate and become post-mitotic before embryonic development completes. The single Cyclin D (*cyd-1)* and CDK4/6 (*cdk-4)* genes are essential for G1/S progression in postembryonic development [12], [13], while the single Rb-family member, *lin-35,* and the *cki-1* and *cki-2* Cip/Kip inhibitors act as negative regulators of cell cycle entry [13]–[18]. Notably, *lin-35* Rb inactivation combined with *cki-1/cki-2* inhibition causes only limited over-proliferation in blast-cell lineages ([13], JK and SvdH, unpublished observations). Thus, in *C. elegans,* cell cycle exit can occur without a functional pRb family protein, as was recently also observed in mice [19].

Here, we examine to what extent terminally differentiated *C. elegans* body-wall muscle cells respond to, or resist, cell-cycle inducing signals. Expression of G1 Cyclin/CDK combinations triggered expression of S phase genes and partial cell cycle re-entry in differentiated muscle cells, while not interfering with the differentiation status. Tissue-specific transcriptional profiling and single molecule FISH experiments revealed that CYD-1/CDK-4 and CYE-1/CDK-2 induce a substantial and overlapping set of genes that are strongly associated with cell cycle functions. However, CYD-1/CDK-4 also triggered up-regulation or down-regulation of large numbers of genes with metabolism-associated functions, and induced DNA replication, in contrast to CYE-1/CDK-2. Notably, several key G1/S regulators, such as *cye-1, cdc-25.1,* and *cdk-2*, were not induced by CYD-1/CDK-4. Thus, differentiated muscle cells remain remarkably flexible for cell cycle induction, while at the same time, robust repression of a few key regulators appears to maintain a stable post-mitotic state.

## Results

### G1 Cyclin/CDK induction overcomes cell cycle quiescence

We set out to examine if cell cycle entry can be induced by transcriptional induction of single G1 Cyclins (CYD-1 Cyclin D or CYE-1 Cyclin E), CDKs (CDK-4, CDK-2, or CDK-2AF, a mutated form of CDK-2 that lacks the negative Tyr14 and Thr15 phosphorylation sites), or combined expression of CYD-1/CDK-4, CYE-1/CDK-2 or CYE-1/CDK-2AF. We first examined the effects of G1 Cyclin/CDK transgene expression in cells that are temporarily arrested (quiescent) but not terminally differentiated. In wild-type larvae that hatch from the egg, precursor cells of the post-embryonic lineages remain quiescent under starvation conditions. When food is added, development resumes and postembryonic blast cells initiate proliferation [14]. Expression of any of the three Cyclin/CDK combinations from the intestine-specific *elt-2* promoter prevented the normal cell cycle arrest in the intestine of late embryonic and starved L1 animals (see Figure S1). Based on BrdU incorporation, DNA replication continued in starvation-arrested larvae with intestinal Cyclin/CDK expression (Figure S1D). The number of intestinal nuclei in these starved L1 animals regularly exceeded the maximal number of 34 nuclei in normal adults (Figure S1B and S1F). Expression of either CYD-1 or CYE-1 alone was sufficient to trigger nuclear division and DNA replication, whereas CDK expression alone did not induce an apparent cell cycle response (Figure S1E and S1F; and data not shown). This probably indicates that the temporally arrested cells contain residual CDK proteins, but not G1 Cyclins. We conclude that transcriptional induction of a G1 Cyclin is sufficient to prevent cell cycle quiescence of intestinal cells.

### Cyclin/CDK expression in muscle leads to cell cycle re-entry during larval development

**Figure 1:**
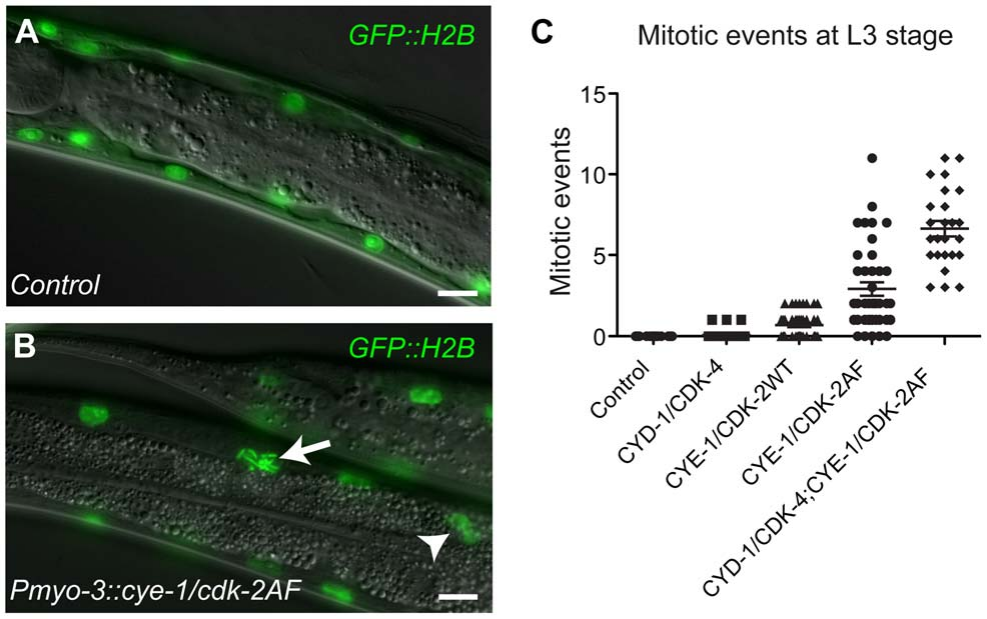
G1 Cyclin/CDK expression induces mitotic events in body-wall muscle cells. (A) Control animal expressing the GFP::H2B fusion protein in body-wall muscle. (B) Animals that express CYE-1, CDK-2AF and GFP::H2B together from the *myo-*3 promoter show chromosome condensation (arrow) and nuclear division (arrowhead) in differentiated body-wall muscle. (C) Quantification of mitotic events for each Cyclin/CDK combination at the L3 stage. Each dot represents the number of mitotic muscle nuclei (with condensed DNA, metaphase or anaphase figures or nuclear division) in a single animal. In each animal, 58 muscle nuclei anterior of the prospective vulva were counted, to exclude muscle cells formed during post-embryonic development in the Mesoblast lineage. The highest mitotic index was 12% (7/58) in the strain with both Cyclin/CDKs. Error bars represent S.E.M.

Next, we examined if G1 Cyclin/CDK expression could trigger cell cycle re-entry in terminally differentiated body-wall muscle. The *C. elegans* larva is born with 81 fully differentiated body-wall muscle cells [10]. We chose the *myo-3* promoter (*Pmyo-3)* to drive expression of Cyclins and CDKs in muscle, as the muscle myosin gene *myo-3* is turned on in post-mitotic embryonic body-wall muscle [20], [21]. When expressed from this promoter, CYE-1 and CDK-2AF showed muscle-specific expression in immunostaining, and complex formation in immunoprecipitation/western blotting experiments (Figure S2). Next, we introduced the different Cyclin/CDK combinations together with a reporter construct, *Pmyo-3::GFP::H2B,* to facilitate the detection of muscle nuclei, and generated strains with integrated arrays to avoid mosaic expression (Figure 1A).

In contrast to our findings in the intestine, animals expressing any of the three Cyclin/CDK combinations in muscle hatched with a normal complement of muscle nuclei (Figure S3A and S3B). Thus, *Pmyo-3*-driven Cyclin/CDK expression does not lead to extra muscle cell division during embryogenesis. However, from the L2 stage onwards, some body-wall muscle cells started to show signs of mitosis, including chromosome condensation, chromosome congression, anaphase and nuclear division, sometimes even resulting in clusters of small nuclei (Figure 1B; Figure S3C-S3E). In contrast to quiescent intestinal cells, expression of CYD-1 or CYE-1 alone did not induce mitotic events in differentiated muscle cells. CYE-1/CDK-2AF was much more efficient in inducing mitotic events than CYD-1/CDK-4, while CYE-1/CDK-2WT expression gave an intermediate effect (Figure 1C). Western blotting experiments indicated that the extent of mitotic induction did not correspond to the protein expression level, but rather the type of CDK (and Cyclin) expressed (Figure S4). The combination of CYD-1/CDK-4 and CYE-1/CDK-2AF caused the strongest mitotic induction (Figure 1C).

**Figure 2:**
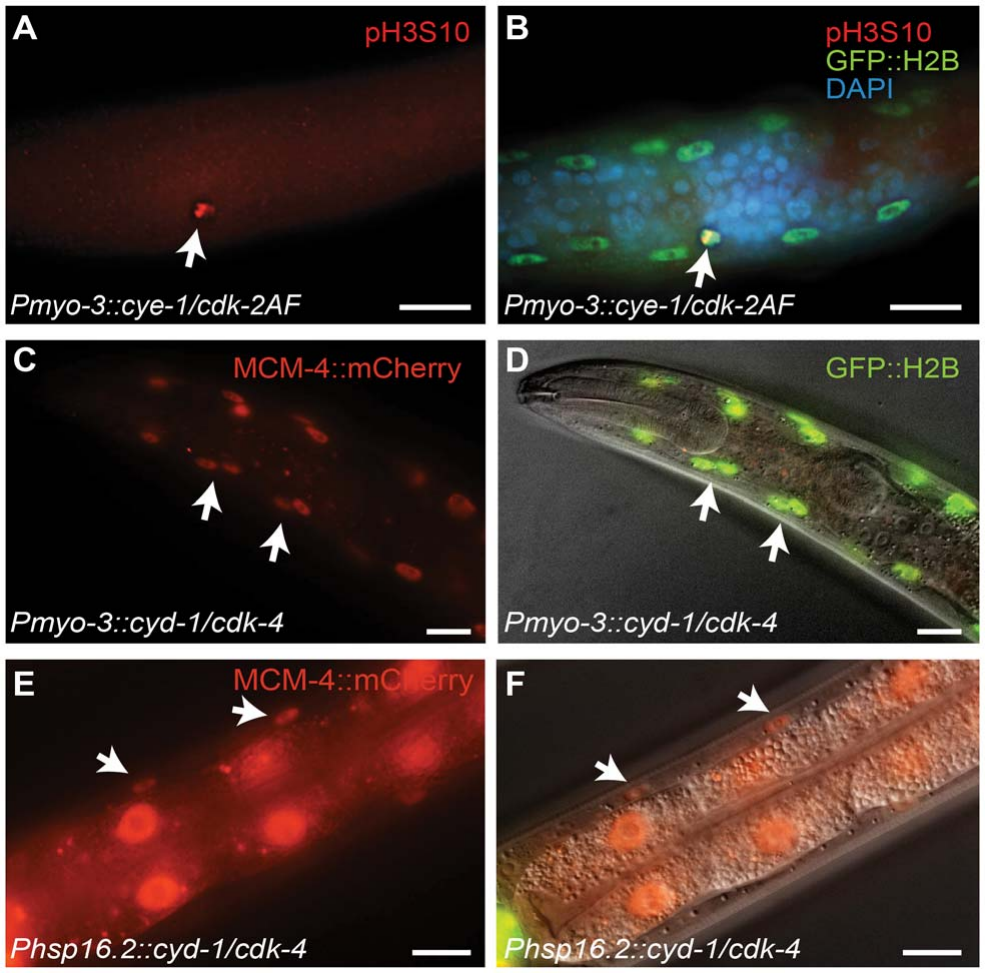
G1 Cyclin/CDK expression induces S and M phase markers in larval muscle. (A,B) A body-wall muscle cell expressing CYE-1/CDK-2AF stains positive for the mitosis-specific phospho-histone H3S10 epitope (arrow). (C,D) Expression of the S-phase marker *Pmcm-4::MCM-4::mCherry* (MCM4) in all body-wall muscle cells of an adult animal expressing CYD-1/CDK-4 from the *myo-3* promoter. (D) merged image of MCM-4::mCherry, GFP::H2B and DIC. (E,F) Expression of the *Pmcm-4::MCM-4::mCherry* S-phase marker in an L4 animal after heat-shock induced expression of CYD-1/CDK-4 at the L2/L3 stage. (F) merged image of MCM-4::mCherry and DIC. Arrows point to muscle nuclei, scale bars indicate 10 μm.

We looked for additional indications of cell cycle re-entry in body-wall muscle cells. Staining for the mitosis specific phospho-histone H3S10 epitope readily visualized mitotic nuclei in the body-wall muscle of CYE-1/CDK-2AF animals (Figure 2A and 2B). Moreover, we observed expression of S-phase reporters in muscle cells. One of these reporters uses the *rnr-1* ribonucleotide reductase promoter to express tdTomato in frame with a destruction box containing N-terminal CYB-1 Cyclin B fragment [22]. In addition, we used a transgenic strain with a single-copy translational fusion of *C. elegans MCM-4::mCherry* under its native promoter. Both reporters were completely silent in body-wall muscle of normal larvae. In contrast, MCM-4::mCherry was detectable as early as the L1 stage in all body-wall muscle cells of CYD-1/CDK-4 and CYE-1/CDK-2AF-expressing animals (Figure 2C and 2D, and data not shown). Expression of *rnr-1::CYB-1desBox::tdTomato* became detectable from the L2 stage onward, and even remained detectable in the muscle of adult animals.

Importantly, heat-shock promoter driven expression of CYD-1 and CDK-4 during larval development also induced S-phase reporter gene expression. After heat shock, we observed MCM-4::mCherry expression in the body-wall muscle of *hsp-16.2::CYD-1/hsp-16.2::CDK-4* transgenic strains (Figure 2E and 2F; in 3 of 3 strains), but never in control heat-shock treated animals without the Cyclin/CDK transgenes (n = 50 animals examined). Even adult animals showed MCM-4 expression after heat-shock induction of CYD-1 and CDK-4, further illustrating that the S phase reporter could be turned on after terminal differentiation. Together, these results indicate that at least some cell cycle genes are not irreversibly silenced in post-mitotic muscle cells.

### *C. elegans* Cyclin D/CDK-4, but not Cyclin E/CDK-2, induces S phase in muscle cells

**Figure 3:**
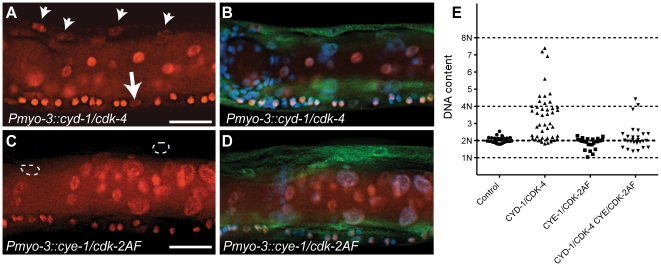
*C. elegans* Cyclin D/CDK-4 induces DNA replication in muscle. (A-D) Detection of EdU incorporation in body-wall muscle nuclei. (A,C) EdU staining, (B,D) merged image of EdU, GFP and DAPI staining. EdU-positive nuclei are readily detectable in CYD-1/CDK-4 expressing body-wall muscle (A, arrowheads), but not in CYE-1/CDK-2AF expressing body-wall muscle (C, circles). For comparison, the arrow in (A) indicates a Pn.p cell, which completed one round of DNA replication in the presence of EdU. (E) Quantitative determination of DNA content reveals DNA replication in CYD-1/CDK-4 expressing muscle cells. Each dot indicates the DNA content, based on propidium iodide staining, of a single body-wall muscle nucleus. Pn.p nuclei in the ventral cord were used as 2n controls. L3/L4 stage larvae were stained in A-D, GFP staining reveals muscle expressed GFP::H2B and CDK-2/4::Venus.

Next, we tested if G1 Cyclin/CDK expression is sufficient for induction of DNA replication in differentiated muscle cells. We used two independent methods to detect DNA synthesis. First, we examined incorporation of EdU, a thymidine analogue that can be used to visualize DNA replication in combination with antibody staining of muscle nuclei [22], [23]. We stained L4 animals for both EdU and GFP and analyzed muscle cells anterior of the vulva, to exclude muscles formed in the postembryonic Mesoblast lineage. To our surprise, only in 2 out of 35 CYE-1/CDK-2AF expressing animals EdU incorporation was detectable in a few muscle cells, while all other muscle nuclei were completely EdU negative (Figure 3C and 3D, circles). In contrast, the anterior of more than half (19/35) of the CYD-1/CDK-4 animals contained clearly EdU positive body-wall muscle cells (Figure 3A and 3B, arrowheads).

Quantitative determination of DNA content based on propidium iodide (PI) intercalation confirmed and expanded these results (Figure 3E). While body-wall muscle in the *Pmyo-3::H2B::GFP* control strain showed a G1 DNA content (2n), muscle cells with CYD-1/CDK-4 expression often contained a larger amount of DNA, which corresponded to a partly or completely duplicated genome (4n). CYE-1/CDK-2 expressing muscle cells did not contain more than 2n DNA, and very few muscle cells with combined expression of CYD-1/CDK-4 and CYE-1/CDK-2 contained 4n DNA (Figure 3E). Thus, CYD-1/CDK-4 promotes DNA replication, while CYE-1/CDK-2 even appears to inhibit induction of DNA synthesis by CYD-1/CDK-4. Together, CYE-1/CDK-2AF expression in differentiated muscle triggers S-phase gene expression and mitosis, but not DNA replication, while the CYD-1/CDK-4 combination induces a more normal cell cycle that includes DNA replication in S phase, but usually arrests prior to M phase.

### Activation of a cell-cycle transcriptional program without loss of muscle differentiation

**Figure 4:**
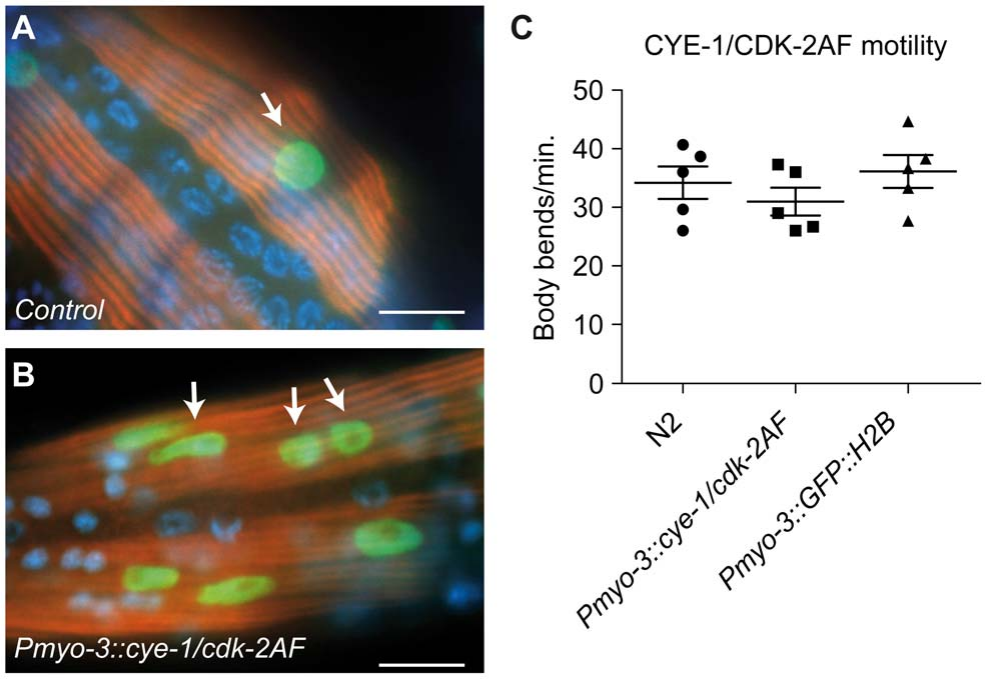
Animals with mitotic body-wall muscle retain normal motility and muscle structure. (A,B) UNC-15/Paramyosin staining (red) of a control (*Pmyo-3::GFP::H2B*) line and a line with muscle expression of CYE-1 and CDK-2AF. Arrows mark GFP (green) expressing nuclei of body-wall muscle cells. (B) Body-wall muscle in both animals show thick filament structures, despite the signs of mitosis (arrows). (C) Motility assay of L4 larvae: N2 wild type, SV858 (*Pmyo-3::CYE-1/CDK-2AF*) and SV859 (*Pmyo-3::GFP::H2B* control). Each dot represents a single animal. Error bars represent S.E.M., scale bars indicate 10 μm.

Our combined data indicate that differentiated body-wall muscle cells can re-enter the cell cycle post-embryonically in response to G1 Cyclin/CDK expression. Next, we wanted to examine if the observed cell cycle re-entry coincides with loss of muscle differentiation. Animals expressing G1 Cyclin/CDK complexes in the body-wall muscle appear phenotypically normal and show apparently normal sinusoidal movement (Figure 4C). To examine muscle structure, we stained animals for UNC-15/Paramyosin, a component of thick muscle filaments in *C. elegans* [24]. Muscle cells in L4 and adult CYE-1/CDK-2AF animals displayed a normal pattern of thick muscle filaments, even when nuclei with clear mitotic figures were present (Figure 4A and 4B). These data support the idea that CYE-1/CDK-2AF expression does not change muscle structure and function.

To obtain a more comprehensive picture of the changes induced by CYD-1/CDK-4 and CYE-1/CDK-2AF expression, we performed tissue-specific mRNA profiling of body-wall muscles. Key to this approach is muscle-specific expression of a FLAG-tagged PAB-1 poly(A)-binding protein [25]. As a validation of the method, immunopurification of PAB-1-crosslinked mRNA from *Pmyo-3::FLAG::PAB-1* transgenic animals yielded mRNAs that were highly enriched for muscle-expressed genes (Table S1). We next compared the mRNA profiles of control L1 animals (carrying the *Pmyo-3::GFP::H2B* and *Pmyo-3::FLAG::PAB-1* transgenes), and L1 animals that, additionally, expressed CYE-1/CDK-2AF or CYD-1/CDK-4 in muscle cells. Most genes in CYD-1/CDK-4 or CYE-1/CDK-2AF expressing muscle cells were not significantly up- or down- regulated (Figure S5A and S5B, Table S2). This includes nearly all muscle-specific genes, confirming maintenance of the muscle-specific fate (see below).

**Figure 5:**
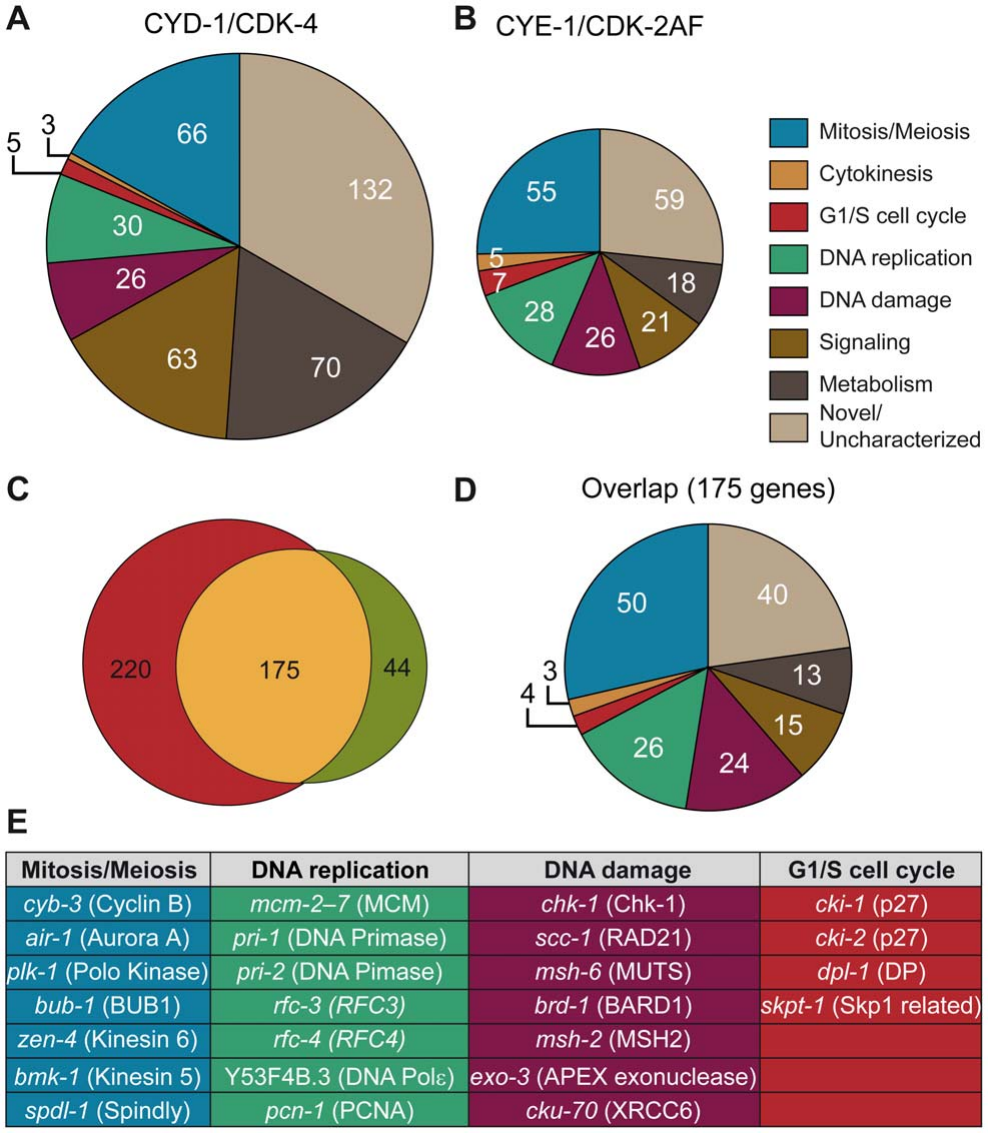
Microarray analysis reveals induction of cell-cycle gene transcripts in differentiated muscle cells. (A,B) Functional annotation of genes significantly increased more than 2-fold in *Pmyo-3::CYD-1/CDK-4* and *Pmyo-3::CYE-1/CDK-2AF* muscle cells. A large overrepresentation of genes involved in various stages of the cell cycle is seen in both data sets, as well as a distinct increase in the proportion of metabolism and signaling genes in CYD-1/CDK-4 animals. (C) Overlap in more than 2-fold upregulated genes between CYE-1/CDK-2AF and CYD-1/CDK-4 body-wall muscle cells. The majority of the genes induced by CYE-1/CDK-2AF are also induced in CYD-1/CDK-4, but the latter also contains a substantial number of non-cell cycle genes. (D) Functional annotation of the genes present in the overlap between CYD-1/CDK-4 and CYE-1/CDK-2AF reveals a strong (60%) representation of known cell cycle genes. (E) Table of representative cell cycle genes and their orthologs that are induced by both CYD-1/CDK-4 and CYE-1/CDK-2AF (>2-fold upregulated).

A set of 219 genes was significantly upregulated (fold-change ≥ 2, p<0.05) in CYE-1/CDK-2AF animals compared to the control strain. Manual annotation of these induced genes and GO-term enrichment analysis using Funcassociate [26] revealed a large overrepresentation of genes involved in various aspects of the cell cycle, including G1/S regulation, DNA replication, DNA damage response, mitosis, cytokinesis and checkpoint control (Figure 5B and 5E, Table S2). Similar to CYE-1/CDK-2AF, CYD-1/CDK-4 expression in the body-wall muscle induced a set of 395 genes with a strong cell cycle signature (Figure 5A, Table S2).

The majority of genes upregulated in CYE-1/CDK-2AF were also upregulated in CYD-1/CDK-4 muscle (Figure 5C and 5D). The overlapping gene set has a particularly strong cell cycle signature, with over 60% of the genes with functional annotations in a cell cycle category (Figure 5D and 5E). Interestingly, CYD-1/CDK-4 also induced a set of 143 genes (> 2x, p<0.05) that are not significantly upregulated by CYE-1/CDK-2AF expression (p>0.05) (Table S2, Table S3). These genes were enriched for GO terms related to biosynthesis, though at adjusted P values slightly above 0.05 (0.055 to 0.092). Manual annotation confirmed that many of these genes (45 of 87 genes with recognizable homologues) have ascribed cellular growth and metabolism functions (Figure 5A, Table S2). This suggests that CYD-1/CDK-4 does not only activate a cell-cycle transcriptional program, but also functions in G1 to stimulate cellular growth and metabolism associated gene expression.

A major function of G1/S Cyclin/CDK complexes is the activation of E2F transcription factors through inhibitory phosphorylation of pRb proteins [1]. We therefore analyzed the promoter sequences of genes upregulated in CYD-1/CDK-4 or CYE-1/CDK-2AF for enrichment in consensus transcription factor binding sites (Materials and Methods), including a *C. elegans* specific profile for the EFL-1 (E2F) transcription factor [27]. We analyzed the promoter regions of 439 genes upregulated ≥ 2 fold in CYD-1/CDK-4 or CYE-1/CDK-2AF expressing muscle. In both sets of promoters the highest scoring transcription factor site motif was that of E2F1, while the *C. elegans* specific EFL-1 site ranked fifth and third respectively (Table S4). These results are consistent with the activation of a set of E2F target genes by both CYD-1/CDK-4 and CYE-1/CDK-2. We next analyzed the 143 genes upregulated in CYD-1/CDK-4 animals but not in CYE-1/CDK-2 animals. This set of genes was hardly enriched for E2F1 and EFL-1 sites (Table S4). Together with the functional annotations, these results indicate that CYD-1/CDK-4 and CYE-1/CDK-2 induce a core set of E2F regulated cell cycle genes, while CYD-1/CDK-4 also activates a set of genes with a broader range of functions.

CYE-1/CDK-2 expression in muscle caused down-regulation of only 50 genes with no functional enrichment. In contrast, CYD-1/CDK-4 expression also resulted in down-regulation (≥ 2x, p<0.05) of a substantial set of 555 transcripts (Table S5). This group shows a remarkable enrichment with GO terms related to catabolic processes, including peptidase, lipase, esterase and hydrolase activities. While muscle cells remain functional and morphologically normal, 60 of the down-regulated genes are normally highly expressed in muscle (Table S4). Thus, CYD-1/CDK-4 causes upregulation of cell-cycle and biosynthesis-associated genes, as well as down-regulation of genes primarily associated with biodegradation and energy production.

### Key G1/S regulators are not induced by G1 Cyclin/CDK expression in muscle

Despite the robust induction of many E2F targets, some key activators of G1/S progression were not induced. For instance, in CYD-1/CDK-4 expressing muscle, the induction factor (log_2_, mean of 4 experiments) was 0.11 for *cye-1* Cyclin E, 0.04 for *cdk-2,* and -1.3 for *cdc-25.1*. These genes are well-established E2F targets in mammals, *Drosophila,* and, at least *cye-1* and *cdc-25.1,* also in *C. elegans* [27], [28]. These observations suggest that in contrast to many other E2F targets, the actual regulators that promote cell cycle entry may be more tightly repressed.

**Figure 6:**
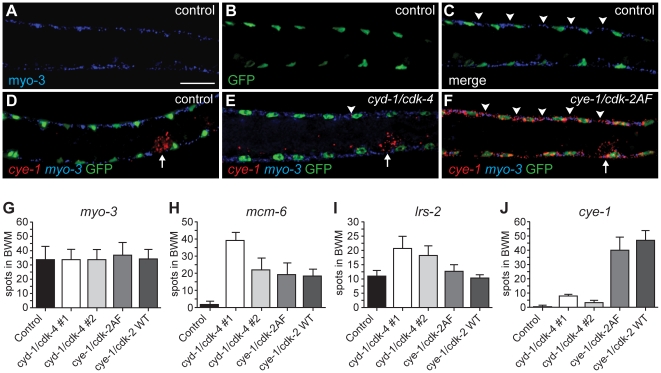
Single molecule FISH shows gene expression in individual muscle cells and limited *cye-1* induction by CYD-1/CDK-4. (A-F) Single mRNA molecules labeled with probes against *myo-3* (blue) or *cye-1* (red) mRNA. Muscle nuclei are marked in green by *myo-3::H2B::GFP*. Analyzed are strains expressing only *Pmyo-3::H2B::GFP* (control, SV859: A-D), and *Pmyo-3::H2B::GFP* together with *Pmyo-3::CYD-1/CDK-4* (SV857: E) or *Pmyo-3::CYE-1/CDK-2AF* (SV858: F). Arrowheads indicate areas of *myo-3* mRNA (C) or *cye-1* mRNA (E, F) expression in dorsal body-wall muscle cells. Arrows in (D-F) indicate *cye-1* expression in the gonad. Scale bar indicates 10 µm, all panels show the same magnification. (G-J) Quantification of the number of fluorescent spots in seven body-wall muscle cells located anterior of the gonad on the dorsal side of animals. All animals were hybridized with *myo-3* probes to identify body-wall muscle cells. Signal corresponding to mRNA molecules of *myo-3* (G), *mcm-6* (H), *lrs-2* (I), or *cye-1* (J) were counted. The strains analyzed are SV859 (control), SV857 (*cyd-1/cdk-4 #1*), SV860 (*cyd-1/cdk-4 #2*), SV858 (*cye-1/cdk-2AF*) and SV861 (*cye-1/cdk-2WT*). Note that overlap between spots greatly reduced the counted number of *myo-3* and *mcm-6* mRNAs. In contrast, the few and small *cye-1* labeled spots are likely all single mRNA molecules.

We used single molecule FISH analysis to verify the gene expression analysis. This method makes use of approximately 48 individually labeled short oligonucleotide probes, which together allow detection of individual mRNA molecules [29]. As a first test, we examined *myo-3* mRNA abundance. The control strain, expressing *myo-3::H2B::GFP* in muscle, and four different strains with muscle-induced G1 Cyclin/CDK expression, all showed readily detectable fluorescent *myo-3* mRNA spots in the cytoplasm of muscle cells (Figure 6A–6C, 6G). Very similar numbers were obtained in different strains and experiments; illustrating that single molecule FISH gives reproducible results (Figure 6G, and data not shown). Next, we used this technique to examine mRNA levels of the E2F-target *mcm-6.* All muscle cells with G1 Cyclin/CDK expression showed substantial *mcm-6* mRNA induction as early as 3 hrs of postembryonic L1 development, in agreement with the microarray data (Figure 6H). Induction of *mcm-6* was observed in CYE-1/CDK-2AF and CYE-1/CDK-2 expressing strains, and in two independent CYD-1/CDK-4 strains, including one with low levels of CYD-1/CDK-4 expression in muscle (CYD-1/CDK-4 #2; Figure 6H, Figure S4).

The microarray data showed weak upregulation of the Leucyl amino-acyl tRNA synthetase gene *lrs-2* in muscle with CYD-1/CDK-4 (induction factor 1.38) but not in CYE-1/CDK-2AF expressing muscle (induction 0.04). FISH experiments showed the same trend: strain 2 with only low CYD-1/CDK-4 expression showed *lrs-2* upregulation, while even the high CYE-1/CDK-2 expressing strain did not differ from the control (Figure 6I). In contrast, *cye-1* mRNA was nearly absent in CYD-1/CDK-4 expressing muscle cells, with occasionally a single or at most two small dots in muscle cells (Figure 6E, 6J). Thus, the single molecule FISH experiments support our conclusions from the microarray data, and show with single cell resolution that *cye-1* mRNA expression is not even induced in a subset of muscle cells.

## Discussion

In this study, we demonstrate that post-mitotic *C. elegans* muscle cells remain remarkably competent to express cell cycle genes, and that cell cycle re-entry can occur coincident with the differentiated state. In addition, we demonstrate differential activity of CYD-1/CDK-4 versus CYE-1/CDK-2 in the regulation of gene expression. These results challenge prevailing views on the irreversible commitment to cell cycle exit upon terminal differentiation and the linear pathway of CDK activities.

To our knowledge, our data show for the first time that the CYD-1/CDK-4 complex controls expression of metabolic genes, in addition to cell cycle genes. These transcriptome data fit well with observations that Cyclin D is directly controlled by mitogenic signals as the most upstream regulator in cell cycle entry (reviewed in [30]), and that *Drosophila* Cyclin D and Cdk4 predominantly act to promote growth rather than cell cycle progression [31], [32]. While many studies implicate Cyclin D and Cyclin E CDKs as regulators of the Rb/E2F module, our data provide strong support for CYD-1/CDK-4 control of additional transcription factors. Interestingly, Cyclin D has been observed to interact with several transcriptional regulators, to localize to promoters, and to affect transcription independently of CDK4/6 (*e.g.,* [33]). It will be of great interest to find if these results relate to our observations, and what molecular mechanisms underlie the regulation of metabolic genes by Cyclin D/CDK4. As a potential substrate, several metabolic genes that are specifically up-regulated (*e.g., acs-2*) or down-regulated (*e.g., ech-9*/F01G10.3) by CYD-1/CDK-4 are transcriptional targets of NHR-49, a member of the Hepatocyte Nuclear Factor 4 (HNF4) family of nuclear hormone receptors [34].

CYD-1/CDK-4 also induced DNA replication in muscle, while CYE-1/CDK-2, in contrast, appeared to antagonize DNA synthesis. As a possible explanation, DNA synthesis might depend on a growth-related signal that is uniquely induced by CYD-1/CDK-4. Alternatively, or in addition, the different effects of the two Cyclin/CDK combinations may result from different roles in DNA replication origin licensing. CYE-1/CDK-2 antagonizes the formation of DNA pre-replication complexes (pre-RCs), at least in some cell types [35]-[37]. This activity helps to restrict DNA replication to only once per cell cycle. The observed induction of DNA replication in muscle cells indicates that CYD-1/CDK-4 does not share this CYE-1/CDK-2 function, but allows the formation and function of pre-RCs. In arrested intestinal cells, CYE-1 Cyclin E was sufficient to induce DNA replication, which points to an important difference in the arrested state of quiescent versus post-mitotic cells.

Progression from temporary arrest to irreversible cell cycle exit is thought to depend on epigenetic silencing of cell cycle promoters (*e.g.,* [3]). Our transcriptional profiling indicates that such a repressive chromatin state can be reversed at many cell cycle promoters. As Cyclin/CDK expression does not appear to induce full cell division of body-wall muscle, additional safeguards probably inhibit proliferation. Our expression studies indicate that these safeguards focus on a limited number of critical regulators. Interestingly, expression of E2F in differentiating *Drosophila* eye and wing cells, and differentiation of mouse Rb-family triple knockout cells have also revealed additional levels of control that impinge on Cyclin E during terminal differentiation [19], [38].

Coincident differentiation and proliferation has been observed in some situations, such as proliferating horizontal interneurons in mouse retinoblastoma, proliferating pRb(-/-) hair cells of the mouse inner ear, and *Drosophila* wing hairs and eye cells expressing E2F and Cyclin E [6]-[8]. Our preliminary results indicate that CYD-1/CDK-4 expression also triggers S phase gene expression in differentiated *C. elegans* neurons (data not shown). Together, the different examples indicate potential for finding a common set of gene alterations that can induce cell proliferation in terminally differentiated cells. Despite the success of induced pluripotent stem cells, this could ultimately provide an attractive regeneration strategy, in particular if limited proliferation could be achieved without genome-wide remodeling of the chromatin and loss of the differentiated state.

## Materials and Methods

### Strains, molecular cloning, and transgenes

All strains and culture conditions are listed in Text S1. Expression constructs for *cye-1*, *cyd-1*, *cdk-4*, *cdk-2*, *cdk-2AF,* and GFP::H2B were created using a 2.4 Kb *myo-3* promoter (PCGS1 (*Pmyo-3)*, body-wall muscle expression) or a 5 kb *elt-2* promoter (intestinal expression [39]). Coding sequences of Venus YFP with *C. elegans*-optimized codons (a kind gift of Yuichi Iino) were inserted before the translational stop in all CDK constructs. The CDK-2AF mutant was created by mutating two conserved Wee1 phosphorylation sites (Thr25-to-Ala and Tyr26-to-Phe) by site-directed mutagenesis. *Pmcm-4::MCM-4::mCherry::mcm-4 3′UTR* was recently described [22] and integrated as a single copy using the MosSCI technique [40]. The *Prnr::CYB-1DesBox::TdTomato* marker expresses an N-terminal 100 amino acid part of *C. elegans* CYB-1 (N-CYB-1), which harbors a KEN destruction box sequence for recognition by the APC/C coupled to the TdTomato fluorophore [22]. Multiple transgenic lines were analyzed for each Cyclin/CDK combination. Representative lines with 40-70% F2 transmission were selected for γ-irradiation. Integrated lines were backcrossed with N2 a minimum of 4 times before analysis.

### Immunostaining and detection of DNA replication

For immunostaining of larval stages, animals were fixed in methanol (5 min. at −20°C) and acetone (20 minutes at −20°C) according to [22]. BrdU and EdU staining were performed as described [22], [41]. Primary antibodies used: rabbit anti-phospho-H3S10 (1∶200, Abcam), mouse anti-GFP (1∶100, Sigma), mouse anti-CYE-1 (E. Kipreos, 1∶200), rabbit anti-GFP (1∶100, Molecular Probes), mouse monoclonal antibody 5-23 to UNC-15 (1∶3, tissue supernatant, Developmental Studies Hybridoma Bank). Secondary antibodies used: Donkey anti-mouse FITC or TexasRed and Donkey anti-rabbit FITC or TexasRed (1∶200, Jackson Immunolaboratories).

Quantitative DNA measurements were performed as previously described [41]. In short, series of Z-sections were taken of propidium iodide-stained animals with a confocal scanning laser microscope, and pixel intensities of all sections were added and recalculated to DNA content, using Pn.p ventral cord precursor nuclei as a 2n DNA standard.

### Tissue-specific microarray analysis

Tissue-specific mRNA was isolated from synchronized L1 larvae fed for 3 hours by purification of the poly-A binding protein FLAG::PAB-1 specifically expressed in body-wall muscle cells of L1 larvae [25]. Poly(A)^+^ RNA was isolated essentially as described [25]. Four independently grown biological samples were used for each different line: SV912 (control), SV911 (*CYE-1/CDK-2AF*), and SV985 (*CYD-1/CDK-4*). Array data is submitted to ArrayExpress accession no.: E-TABM-886. Procedures used in the hybridizations, data analysis and transcription factor analysis are detailed in Text S1.

### Functional analysis

Manual annotation of genes upregulated >2-fold was done using WormBase (release WS207). GO-term enrichment was determined using Funcassociate [26].

### Single-molecule Fluorescence In Situ Hybridization

Single molecule Fluorescence In Situ Hybridization was performed as described by [29]. Starved L1 animals were fed for three hours on OP50, followed by fixation in 4% formaldehyde and hybridization with sets of the 48 labeled oligonucleotide probes. Images were taken with a DeltaVision Core wide-field microscope, quantification of the number of mRNAs was performed using the 3D imaging software Volocity version 5 (Perkin Elmer). For quantification, a region of interest was drawn around seven body-wall muscle nuclei, located anteriorly from the gonad on the dorsal side of the animal, to exclude cells in the ventral nerve cord and mesoblast lineage. Further details on the procedure, microscopy and data analysis are provided in Text S1.

## Supporting Information

Figure S1 Expression of G1 Cyclin/CDK combinations in the intestine of arrested L1 larvae leads to extra nuclear divisions and DNA synthesis. (A-B) L1 animals carrying an integrated *Pelt-2::GFP* marker alone (A) or in combination with CYD-1/CDK-4 expressed from the intestinal *elt-2* promoter (B). Arrows indicate clusters of extra nuclei. (C-D) BrdU incorporation in the intestine of wild-type starved L1 control animals (C) or animals expressing CYD-1/CDK-4 in the intestine (D). Control L1 arrested animals have no BrdU positive intestinal cells, only the Q neuroblast daughters (C, brackets) and some epidermal V-cells occasionally escape arrest. The intestine of the *Pelt-2::CYD-1/CDK-4* animal shows an extensive amount of intestinal cells that have undergone DNA replication during starvation induced quiescence (D, arrows). (E) Quantification of the percentage of animals staining positive for BrdU in the gut in representative lines of each Cyclin/CDK combination. (F) Quantification of the number of intestinal nuclei in arrested L1 animals. Note that expression of CYD-1 alone is sufficient to trigger cell-cycle progression in the gut. Each dot represents a single animal. Error bars represent S.E.M.
(TIF)

Figure S2 Expression of CYE-1 and CDK-2AF::Venus in the body wall muscle. A-C: Immunostaining of CYE-1 and GFP in SV858 (*Pmyo-3::GFP::H2B; Pmyo-3::CYE-1/CDK-2AF::Venus*) L1 larva. GFP antibody staining visualizes the body wall muscle nuclei (A), the CYE-1 staining shows nuclear localization of CYE-1 protein in the body wall muscle (B) C: Merge of (A) and (B). D: Immunoprecipitation (IP) of the CYE-1/CDK-2AF::Venus complex. CYE-1 migrates with an apparent molecular weight of ∼72 kDa. The CDK-2AF::Venus fusion protein is detected at 66 kDa with anti-GFP antibodies. The CYE-1/CDK-2AF::Venus interaction was detected in both the CYE-1 and GFP immunoprecipitations.
(TIF)

Figure S3 Body wall muscle expressing CYE-1/CDK-2AF show mitotic events during larval development. (A) GFP-DIC picture of a starvation-arrested L1 animal expressing CYE-1/CDK-2AF from the *myo-3* promoter. No extra nuclei or mitotic nuclei are observed at this stage. (B) Quantification of muscle nuclei in control animals (expressing only *Pmyo-3:GFP::H2B* in their muscle) and CYE-1/CDK-2AF L1 animals after 24 or 48 hours of L1 arrest. N = 15 animals for each condition. Each dot represents a single animal. Error bars represent S.E.M. (C, D, E) During larval development, mitotic body wall muscle nuclei become apparent. L3 stage animals that express *Pmyo-3::CYE-1, Pmyo-3::CDK-2AF* and *Pmyo-3::GFP::H2B* show DNA condensation (C, D, arrowheads) and abnormal nuclear divisions (D,E, arrows) in differentiated body wall muscle.
(TIF)

Figure S4 Western blot analysis of CDK and H2B expression levels in transgenic strains. Total protein lysates of transgenic animals with muscle expression of GFP::H2B alone (control, SV859), or together with Cyclin D/CDK-4::Venus (SV857: *cyd-1/cdk-4* #1, SV860: *cyd-1/cdk-4* #2) or Cyclin E/CDK-2::Venus (SV858: *cye-1/cdk-2AF*, SV861: *cye-1/cdk-2WT*) were separated by SDS PAGE electrophoresis and blotted. The upper panel was probed with an anti-GFP antibody and shows CDK-2/4::Venus (70 and 66 kDa resp., upper arrow) and GFP::H2B (41.5 kDa, lower arrow) protein bands. The lower panel contains the same samples, probed with anti-α-Tubulin (55 kDa) as a loading control (arrow points to α-Tubulin). Note that the *cyd-1/cdk-4* #1 strain shows higher levels of transgene expression than the *cye-1/cdk-2AF* strain. These strains were used in all subsequent experiments, except for *cyd-1/cdk-4* #2, which was only used in the experiments shown in Figure 6.
(TIF)

Figure S5 Scatterplot representation of expressed genes. (A,B) Microarray signal intensities for CYE-1/CDK-2AF (A) and CYD-1/CDK-4 (B) expressing muscle compared to control muscle IP (SV912). The experiment was repeated four times for each line. The intensities of all genes are shown after background subtraction, normalization, and merging of replicate culture dye-swap hybridizations. MAANOVA statistical analysis was performed to determine genes with significantly different mRNA expression. White data points mark genes that are significantly changed (p<0.05) and have a ≥2-fold change. Values are plotted on a log_10_ scale. Y-axis: CYD-1/CDK-4 (SV985) or CYE-1/CDK-2AF (SV911) PAB-1 IP RNA *versus* total RNA, X-axis: Control (SV912) PAB-1 IP RNA *versus* total RNA. (C) Plot of significantly changed genes (p≤ 0.05) in CYE-1/CDK-2AF (Y-axis) and CYD-1/CDK-4 (X-axis). Colors for each data point indicate in which set(s) the gene is significantly changed (green: CYD-1/CDK-4, red: CYE-1/CDK-2AF). Values are plotted on a log_2_ scale.
(TIF)

Table S1 List of genes enriched ≥2-fold in muscle (PAB-1 IP *versus* total RNA) and GO-term analysis of these genes. The experiment was repeated four times for each line. The intensities of all genes are shown after background subtraction, normalization, and merging of replicate culture dye-swap hybridizations. MAANOVA statistical analysis was performed to determine genes with significantly different mRNA expression.
(XLSX)

Table S2 Lists of genes enriched ≥2-fold in CYD-1/CDK-4, CYE-1/CDK-2AF, both, or exclusively in one of the strains, including GO-term analysis and functional classification. This file also includes fold-change values for all significantly changed genes.
(XLSX)

Table S3 Fold change of all probes enriched in CYD-1/CDK-4 or CYE-1/CDK-2AF lines (p-value < 0.05).
(XLSX)

Table S4 Transcription-factor binding sites enriched in the promoters of Cyclin/CDK-induced genes. Includes sequence logos for the recognition sites of the transcription factors identified in the promoter analysis. The Clover raw score indicates how frequently a motif is present. For E2F sites, this score is 44 for the set of 143 genes upregulated in CYD-1/CDK-4 animals but not in CYE-1/CDK-2 animals, as compared to 167 ± 7 on average for a random selection of 143 genes induced by CYD-1/CDK-4 as well as CYE-2/CDK-2AF.
(XLSX)

Table S5 Lists of genes downregulated ≥2-fold in CYD-1/CDK-4, and genes upregulated ≥2-fold in control muscle cells that are downregulated ≥2-fold in CYD-1/CDK-4. Includes GO-term analysis of these genes.
(XLSX)

Text S1 Supplementary Methods, Supplementary References.
(DOC)
