# Supplementary figures and images for: Caenorhabditis elegans Cyclin D/CDK4 and Cyclin E/CDK2 Induce Distinct Cell Cycle Re-Entry Programs in Differentiated Muscle Cells
Source: PLoS Genet. 2011 Nov 10;7(11):e1002362. doi: 10.1371/journal.pgen.1002362 (PMC3213155; doi:10.1371/journal.pgen.1002362)

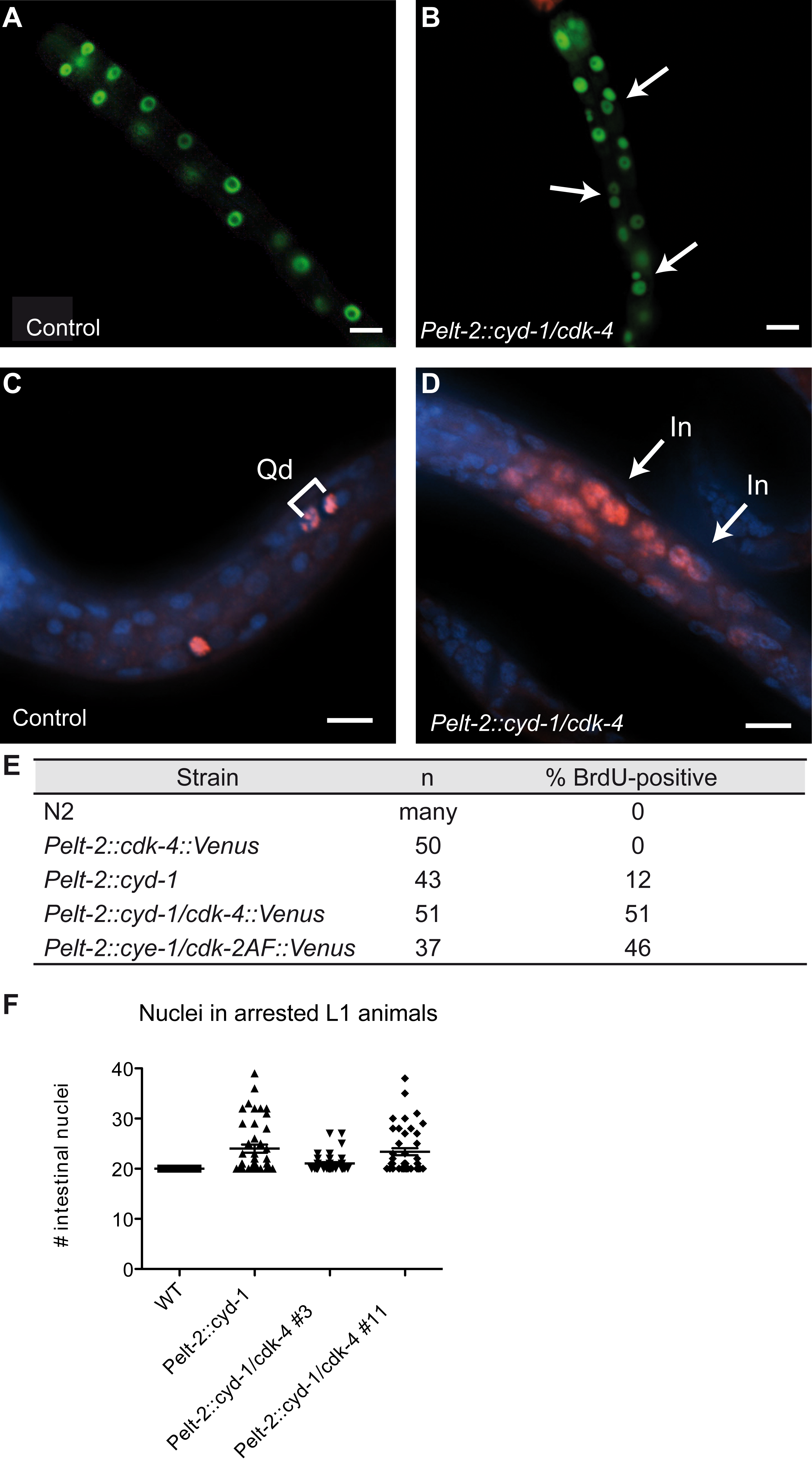

Supplement: Figure S1 — Expression of G1 Cyclin/CDK combinations in the intestine of arrested L1 larvae leads to extra nuclear divisions and DNA synthesis. (A-B) L1 animals carrying an integrated Pelt-2::GFP marker alone (A) or in combination with CYD-1/CDK-4 expressed from the intestinal elt-2 promoter (B). Arrows indicate clusters of extra nuclei. (C-D) BrdU incorporation in the intestine of wild-type starved L1 control animals (C) or animals expressing CYD-1/CDK-4 in the intestine (D). Control L1 arrested animals have no BrdU positive intestinal cells, only the Q neuroblast daughters (C, brackets) and some epidermal V-cells occasionally escape arrest. The intestine of the Pelt-2::CYD-1/CDK-4 animal shows an extensive amount of intestinal cells that have undergone DNA replication during starvation induced quiescence (D, arrows). (E) Quantification of the percentage of animals staining positive for BrdU in the gut in representative lines of each Cyclin/CDK combination. (F) Quantification of the number of intestinal nuclei in arrested L1 animals. Note that expression of CYD-1 alone is sufficient to trigger cell-cycle progression in the gut. Each dot represents a single animal. Error bars represent S.E.M. (TIF) [file pgen.1002362.s001.tif]

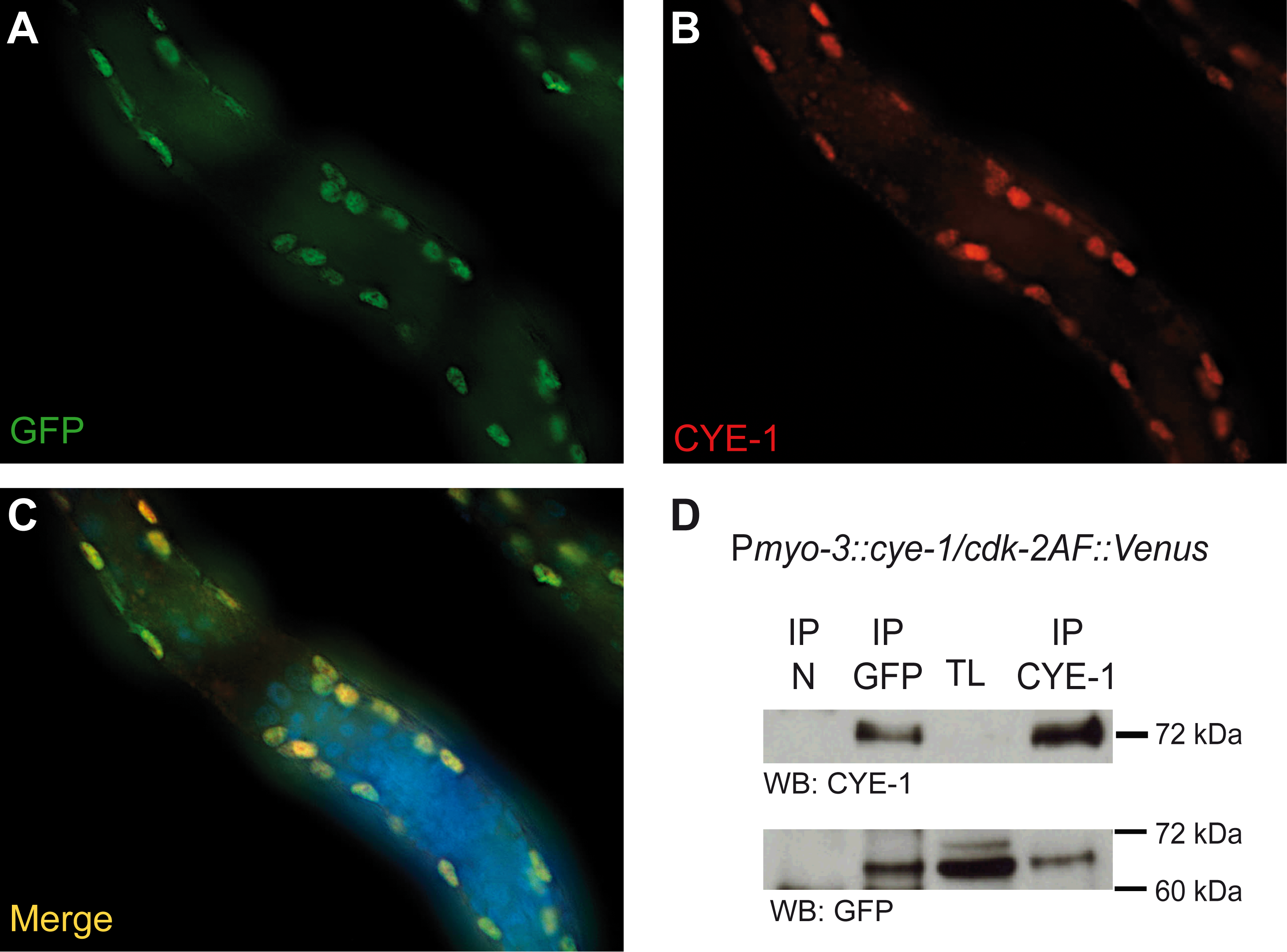

Supplement: Figure S2 — Expression of CYE-1 and CDK-2AF::Venus in the body wall muscle. A-C: Immunostaining of CYE-1 and GFP in SV858 (Pmyo-3::GFP::H2B; Pmyo-3::CYE-1/CDK-2AF::Venus) L1 larva. GFP antibody staining visualizes the body wall muscle nuclei (A), the CYE-1 staining shows nuclear localization of CYE-1 protein in the body wall muscle (B) C: Merge of (A) and (B). D: Immunoprecipitation (IP) of the CYE-1/CDK-2AF::Venus complex. CYE-1 migrates with an apparent molecular weight of ∼72 kDa. The CDK-2AF::Venus fusion protein is detected at 66 kDa with anti-GFP antibodies. The CYE-1/CDK-2AF::Venus interaction was detected in both the CYE-1 and GFP immunoprecipitations. (TIF) [file pgen.1002362.s002.tif]

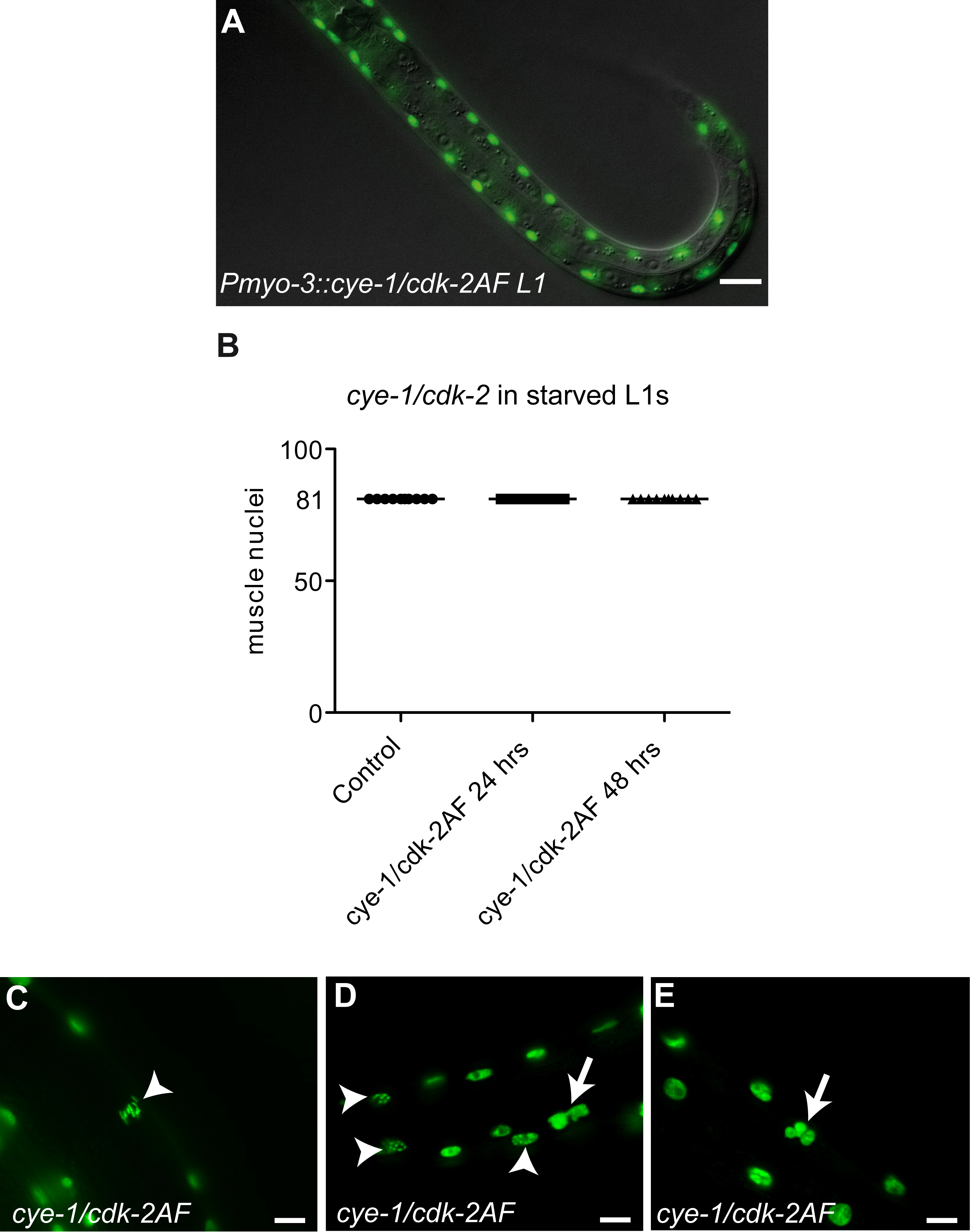

Supplement: Figure S3 — Body wall muscle expressing CYE-1/CDK-2AF show mitotic events during larval development. (A) GFP-DIC picture of a starvation-arrested L1 animal expressing CYE-1/CDK-2AF from the myo-3 promoter. No extra nuclei or mitotic nuclei are observed at this stage. (B) Quantification of muscle nuclei in control animals (expressing only Pmyo-3:GFP::H2B in their muscle) and CYE-1/CDK-2AF L1 animals after 24 or 48 hours of L1 arrest. N = 15 animals for each condition. Each dot represents a single animal. Error bars represent S.E.M. (C, D, E) During larval development, mitotic body wall muscle nuclei become apparent. L3 stage animals that express Pmyo-3::CYE-1, Pmyo-3::CDK-2AF and Pmyo-3::GFP::H2B show DNA condensation (C, D, arrowheads) and abnormal nuclear divisions (D,E, arrows) in differentiated body wall muscle. (TIF) [file pgen.1002362.s003.tif]

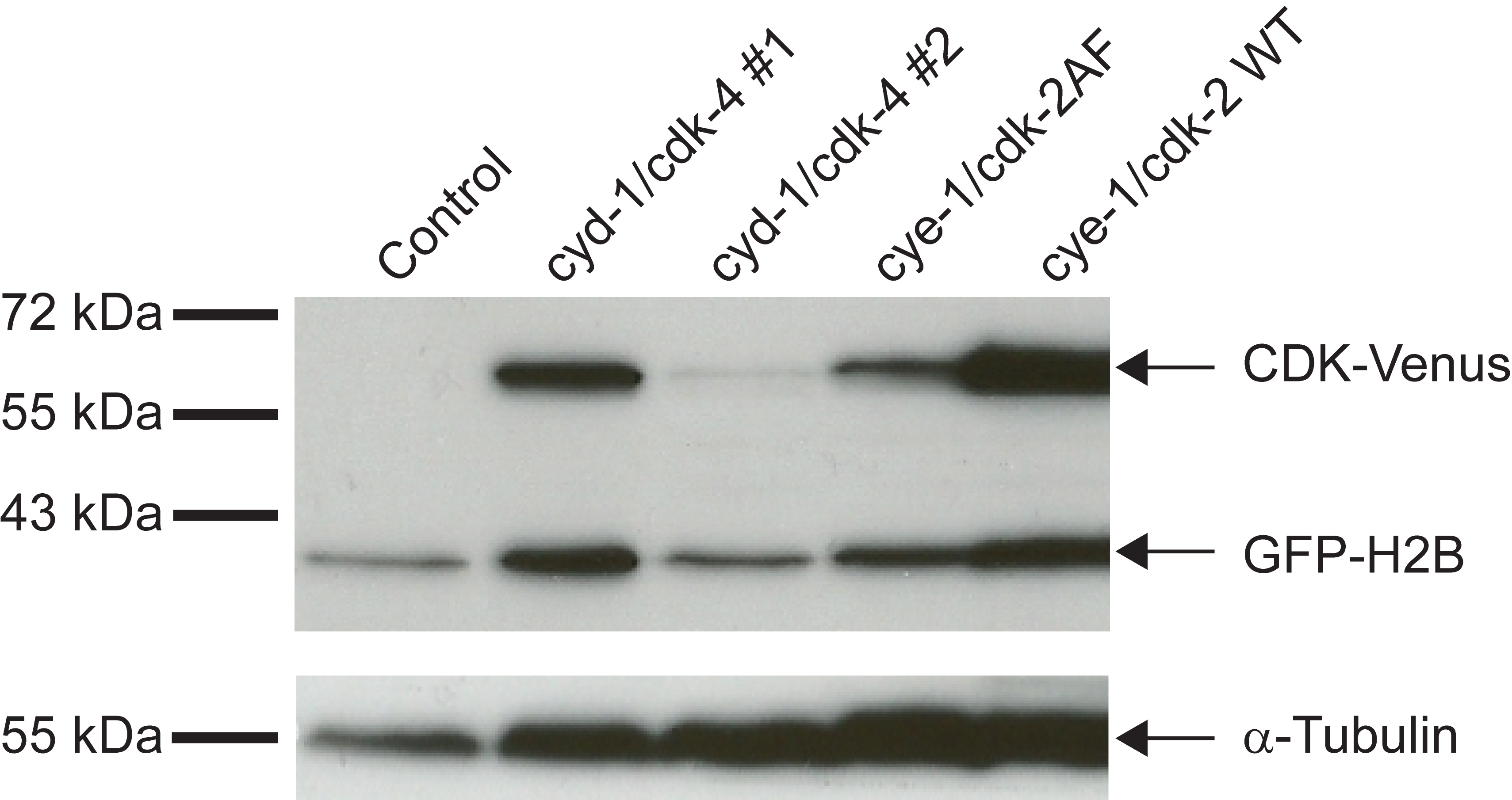

Supplement: Figure S4 — Western blot analysis of CDK and H2B expression levels in transgenic strains. Total protein lysates of transgenic animals with muscle expression of GFP::H2B alone (control, SV859), or together with Cyclin D/CDK-4::Venus (SV857: cyd-1/cdk-4 #1, SV860: cyd-1/cdk-4 #2) or Cyclin E/CDK-2::Venus (SV858: cye-1/cdk-2AF, SV861: cye-1/cdk-2WT) were separated by SDS PAGE electrophoresis and blotted. The upper panel was probed with an anti-GFP antibody and shows CDK-2/4::Venus (70 and 66 kDa resp., upper arrow) and GFP::H2B (41.5 kDa, lower arrow) protein bands. The lower panel contains the same samples, probed with anti-α-Tubulin (55 kDa) as a loading control (arrow points to α-Tubulin). Note that the cyd-1/cdk-4 #1 strain shows higher levels of transgene expression than the cye-1/cdk-2AF strain. These strains were used in all subsequent experiments, except for cyd-1/cdk-4 #2, which was only used in the experiments shown in Figure 6. (TIF) [file pgen.1002362.s004.tif]

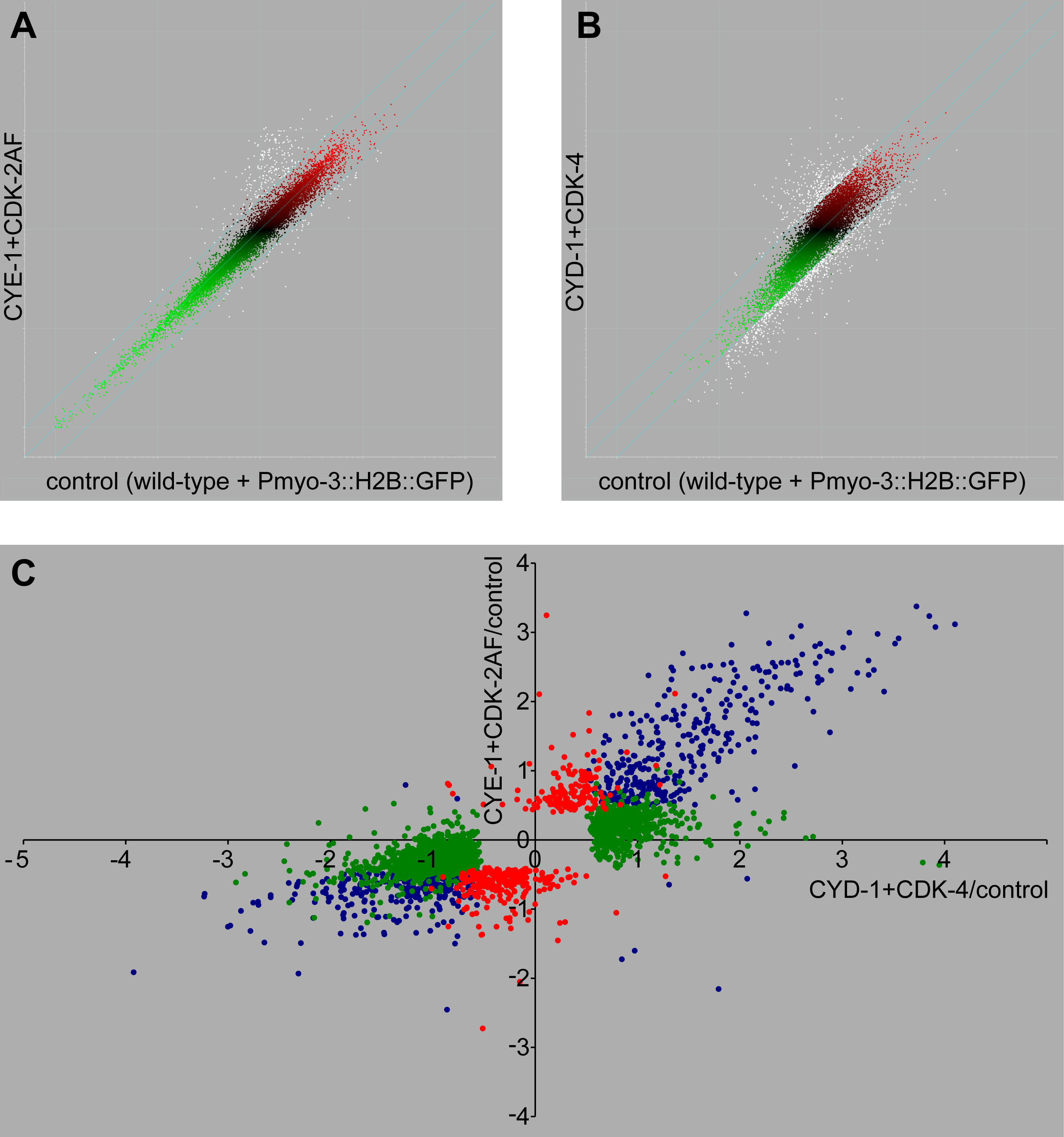

Supplement: Figure S5 — Scatterplot representation of expressed genes. (A,B) Microarray signal intensities for CYE-1/CDK-2AF (A) and CYD-1/CDK-4 (B) expressing muscle compared to control muscle IP (SV912). The experiment was repeated four times for each line. The intensities of all genes are shown after background subtraction, normalization, and merging of replicate culture dye-swap hybridizations. MAANOVA statistical analysis was performed to determine genes with significantly different mRNA expression. White data points mark genes that are significantly changed (p<0.05) and have a ≥2-fold change. Values are plotted on a log10 scale. Y-axis: CYD-1/CDK-4 (SV985) or CYE-1/CDK-2AF (SV911) PAB-1 IP RNA versus total RNA, X-axis: Control (SV912) PAB-1 IP RNA versus total RNA. (C) Plot of significantly changed genes (p≤ 0.05) in CYE-1/CDK-2AF (Y-axis) and CYD-1/CDK-4 (X-axis). Colors for each data point indicate in which set(s) the gene is significantly changed (green: CYD-1/CDK-4, red: CYE-1/CDK-2AF). Values are plotted on a log2 scale. (TIF) [file pgen.1002362.s005.tif]
