# Supplementary material for: Caenorhabditis elegans Cyclin D/CDK4 and Cyclin E/CDK2 Induce Distinct Cell Cycle Re-Entry Programs in Differentiated Muscle Cells
Source: PLoS Genet. 2011 Nov 10;7(11):e1002362. doi: 10.1371/journal.pgen.1002362 (PMC3213155; doi:10.1371/journal.pgen.1002362)
Supplement: Text S1 — Supplementary Methods, Supplementary References. (DOC) [file pgen.1002362.s011.doc]

# *C. elegans* Cyclin D/CDK4 and Cyclin E/CDK2 Induce Distinct Cell Cycle Re-entry Programs in Differentiated Muscle Cells

**Text S1: Supplementary Methods, Supplementary References**

Jerome Korzelius^1^, Inge The^1^, Suzan Ruijtenberg^1^, Martine B. W. Prinsen^1^, Vincent Portegijs^1^, Teije C. Middelkoop^1^, Marian J. Groot Koerkamp^2^, Frank C. P. Holstege^2^, Mike Boxem^1^ and Sander van den Heuvel^1*^

^1^ Developmental Biology, Utrecht University, Padualaan 8, 3584 CH Utrecht, The Netherlands.

^2^ Molecular Cancer Research, University Medical Center Utrecht, Universiteitsweg 100, 3584 CG Utrecht, The Netherlands.

- Correspondence: [S.J.L.vandenHeuvel@uu.nl](mailto:S.J.L.vandenHeuvel@uu.nl)
- +31 302533573/ Fax: +31 302532837

Running Title: Cell cycle re-entry in differentiated cells

# SUPPORTING INFORMATION

# Supplemental Experimental Procedures

## *C. elegans* strains and culturing

*C.* *elegans* strains were cultured on NGM plates seeded with *E. coli* strain OP50 as described [1] at 20°C unless indicated otherwise. For synchronization, animals were treated with hypochlorite solution, followed by hatching of the eggs in M9 medium with 0.05% Tween-20. Larvae were then allowed to develop for the appropriate amount of time.

The following strains were used in this study: N2 Bristol wild-type, SV822 *heEx288[Pelt-2::CYE-1; Pelt-2::CDK-2AF::Venus;Pmyo-2::GFP]*. SV326 *rtIs14[elt-2::GFP; osm-10::HT150Q]*. SV1030 *rtIs14; heEx345[Pelt-2::CDK-4::Venus; Pmyo-2::TdTomato]*. SV1031 *rtIs14; heEx346[Pelt-2::CYD-1; Pelt-2::CDK-4::Venus; Pmyo-2::TdTomato]*. SV857 *heIs9[Pmyo-3::GFP::H2B; Pmyo-3::CYD-1; Pmyo-3::CDK-4::Venus]*. SV858 *heIs10[Pmyo-3::GFP::H2B; Pmyo-3::CYE-1; Pmyo-3::CDK-2AF::Venus].* SV859 *heIs11[Pmyo-3::GFP::H2B].* SV860 *heIs12[Pmyo-3::GFP::H2B; Pmyo-3::CYD-1; Pmyo-3::CDK-4::Venus].* SV861 *heIs44[Pmyo-3::GFP::H2B; Pmyo-3::CYE-1; Pmyo-3::CDK-2::Venus]*. SV1076 *unc-119(ed3); heIs47[Pmcm-4::MCM-4::mCherry]; heIs9*. SV1077 *unc-119(ed3); heIs47; heIs10*. SV852 *heIs10; heIs10[Prnr-1::CYB-1DesBox::TdTomato; pRF4 rol-6(+)]*. SV1118 *unc119(ed3); heIs47; heEx388[Phsp16.2::CYD-1;Phsp-16.2::CDK-4;Pmyo-2::GFP]*. SD1075 *gaIs146[Pmyo-3::FLAG::PAB-1; Psur-5::GFP]*. SV911 *heIs10; gaIs146*. SV912 *heIs11; gaIs146.* SV985 *gaIs146*; *heIs9*. SV1195 *heIs9; heIs10.*

For heat-shock induced expression: synchronized L2/L3 stage SV1118 animals expressing CYD-1/CDK-4 from the *hsp16.2* heat shock promoter were placed for 40 min. at 33^o^C in a water bath and a second heat shock was given 15 hours later. Animals were examined for expression of the S-phase marker *Pmcm-4::MCM-4::mCherry* 8 hours after the second heat shock.

## Microarray Hybridizations and Data Analysis

Microarrays were *C. elegans* Gene Expression Microarrays V1 (Agilent Technologies), with 2x 21000 60-mer probes in a 4x44K layout. RNA amplification, labeling, and hybridizations were performed as described [2,3] with a minimum of 50 ng immunoprecipitated RNA from each sample. After automated data extraction using Imagene 8.0 (BioDiscovery), print-tip loess normalization was performed on mean spot-intensities [4]. Dye bias was corrected with a within-set estimate according to [5]. Statistical analysis was through MAANOVA [6]. In a fixed effect analysis, sample, array, and dye effects were modeled. P-values were determined by a permutation F2-test, in which residuals were shuffled 5000 times globally. Probes with *p<0.05* after family-wise error correction were considered significantly changed. Agilent probe names were matched to gene names from Wormbase release WS200.

## Transcription factor site analysis

To identify transcription factor binding sites enriched in different gene sets we used the Clover program [7] together with the Jaspar CORE set of non-redundant transcription factor binding site profiles (modification date 10-12-2009) [8]. In addition, we added a *C. elegans* specific E2F profile [9]. For each gene we defined the promoter as the sequence from 1000 bp upstream of the first splice variant to the -1 position of the most downstream splice variant. For genes in operons, the promoter sequence of the first gene in the operon was analyzed. To identify absolute E2F transcription site numbers (table S4), we used the TFBS perl module [10]. Sequence logos were drawn using Weblogo [11].

## Motility assays, microscopy and quantification

Motility assays were performed by determining the average number of body bends per minute in a three-minute interval for each animal as described [12]. For UNC-15 Paramyosin staining of L4/adult worms, we used a modified version of the Finney-Ruvkun whole-mount staining protocol [13]. For observation of H2B::GFP localization in live animals, synchronized larvae were anaesthetized with 10 mM Sodium Azide. Extra division events were quantified at L3 and L4 stages by counting the number of cells that had mitotic phenotypes (including cells with extra nuclei, nuclei with condensed DNA, and nuclei in the various stages of mitosis) anterior of the prospective vulva. Photographs were taken with an Axioplan 2 microscope mounted with an Axiocam mRM camera (Zeiss Microscopy). Scalebars are 10 μm. Quantitive data and graphs were produced using GraphPad Prism Version 5 for Mac (GraphPad Software) and Excel 2008 for Mac (Microsoft).

## Co-immunoprecipitation of CYE-1/CDK-2AF::VENUS complex

For co-immunoprecipitation of the CYE-1/CDK-2AF::Venus complex, N2 and SV837 (see *C. elegans* strains and culturing) animals were homogenized by grinding in liquid nitrogen and transferring the worm debris into 4 ml chilled lysis buffer (20 mM Tris-HCl (pH 8.3), 137 mM NaCl, 1% Nonidet P-40, 2 mM EDTA, protease inhibitor cocktail (Roche Diagnostics), 0.14% β-mercapto-ethanol), followed by treatment of the lysate in a French pressure cell. 1 mg of protein was used for the IP experiments and 50 µg was used for direct lysate samples. Immunoprecipitation was performed by incubating 500 µl lysate with either anti-CYE-1 (a kind gift of Michael Krause) or anti-GFP (Molecular Probes) covalently coupled to ProtG and ProtA beads respectively. Negative control immunoprecipitations were performed with mouse anti-SD15 (a kind gift of Nick Dyson), 5 µl of ascitus coupled to protG beads, and rabbit anti-eIF-4E (a kind gift of Adri Thomas) coupled to protA beads. Following immunoprecipitation, 20 µl of each sample was loaded on an 8% polyacrylamide gel. Proteins were blotted on PVDF membrane (Amersham Bioscience). Blots were incubated with mouse anti-CYE-1 (M.Krause, 1:750), rabbit anti-GFP (Invitrogen,1:500) or monoclonal mouse anti-actin (MP Bio Medicals, 1:1000). Goat anti-mouse-peroxidase and goat anti-rabbit peroxidase (Jackson Immunolaboratories) were used as secondary antibodies. The chemiluminescent detection reaction was performed using a Biorad ECL kit (Biorad) and the blot was subsequently exposed to hypersensitive film (Amersham Hyperfilm, GE healthcare).

## Single molecule Fluorescence In Situ Hybridization

Single molecule Fluorescence In Situ Hybridization was performed as described in [14]. In brief, 48 oligonucleotides probes directed against the coding sequence of *myo-3*, *mcm-6*, *lrs-2* and *cye-1* were designed on the single molecule FISH website (www.singlemoleculefish.com) and ordered with 3’-modifications from BioSearch Technologies. All 48 oligonucleotides were pooled and labeled with succinimidyl esters of either Tetramethylrhodamine (TMR) (Invitrogen) or Cy5 (GE Healthcare) and purified by reverse phase liquid chromatography using a C18 polymeric reversed phase column (VYDAC 218TP™ Series). Starved L1 animals were fed for three hours on OP50, followed by fixation in 4% formaldehyde and hybridized with the lableled 48 nucleotides probes. Hybridization of these animals was performed in 100 µl hybridization solution to which 9.3 ng of the *myo-3* probes coupled to TMR was added, and 3.7 ng of *mcm-6*, 18.4 ng of *lrs-2* or 8.6 ng of *cye-1* probes coupled to Cy5. Following overnight incubation at 32ºC, samples were washed 4x 1 hour with wash buffer at 32ºC. Shortly before imaging, samples were mounted in anti-bleach buffer.

## Western blot analysis

Western blotting experiments were performed as previously described [15]. In short: animals from various strains were grown and equal numbers of L4 animals (80) were harvested. 2x Laemmli sample buffer was added to the worm pellet, samples were boiled for 10 minutes, divided in two and separated on a 12% SDS-PAGE gels. Blots were probed with rabbit anti-GFP (Abcam, 1:1000 dilution), and mouse anti-α-tubulin (Sigma, 1:1000 dilution).

## Imaging and quantification

Images were taken with a DeltaVision Core wide-field microscope, equipped with a 100x oil-immersion objective (Applied Precision) and a Cascade 2 EMCCD® 1K camera (Photometrics) using SoftWorx acquisition software version 4 (Applied Precision). Exposure times used for both TMR and Cy5 were 2 seconds. Stacks were acquired automatically with 0.20 microns between the Z-slices.

Quantification of the number of mRNAs was performed using the 3D imaging software Volocity version 5 (Perkin Elmer). A region of interest was drawn around seven body wall muscle nuclei, located anteriorly from the gonad on the dorsal side of the animal, to exclude cells in the ventral nerve cord and mesoblast lineage. Expression of *myo-3* mRNA in the cytoplasm was used to localize the body wall muscle cells. The results were statistically analyzed with GraphPad Prism version 5 (GraphPad Software Inc), using an unpaired t-test.

# Supplemental References

1. Stiernagle T (2006) Maintenance of *C. elegans*. *WormBook*:1-11.

2. van de Peppel J*, et al.* (2003) Monitoring global messenger RNA changes in externally controlled microarray experiments. *EMBO Rep* 4(4):387-393.

3. Roepman P*, et al.* (2005) An expression profile for diagnosis of lymph node metastases from primary head and neck squamous cell carcinomas. *Nat Genet* 37(2):182-186.

4. Yang YH*, et al.* (2002) Normalization for cDNA microarray data: a robust composite method addressing single and multiple slide systematic variation. *Nucleic Acids Res* 30(4):e15.

5. Margaritis T*, et al.* (2009) Adaptable gene-specific dye bias correction for two-channel DNA microarrays. *Mol Syst Biol* 5:266.

6. Wu H, Kerr MK, Cui X, & Churchill GA (2002) MAANOVA: a software package for the analysis of spotted cDNA microarray experiments. *The analysis of gene expression data: methods and software*:313-341.

7. Frith MC*, et al.* (2004) Detection of functional DNA motifs via statistical over-representation. *Nucleic Acids Res* 32(4):1372-1381.

8. Portales-Casamar E*, et al.* (2009) JASPAR 2010: the greatly expanded open-access database of transcription factor binding profiles. *Nucleic Acids Res* .

9. Kirienko NV & Fay DS (2007) Transcriptome profiling of the *C. elegans* Rb ortholog reveals diverse developmental roles. *Dev Biol* 305(2):674-684.

10. Lenhard B & Wasserman WW (2002) TFBS: Computational framework for transcription factor binding site analysis. *Bioinformatics* 18(8):1135-1136.

11. Crooks GE, Hon G, Chandonia JM, & Brenner SE (2004) WebLogo: a sequence logo generator. *Genome Res* 14(6):1188-1190.

12. Robatzek M & Thomas JH (2000) Calcium/calmodulin-dependent protein kinase II regulates *Caenorhabditis elegans* locomotion in concert with a G(o)/G(q) signaling network. *Genetics* 156(3):1069-1082.

13. Bettinger JC, Lee K, & Rougvie AE (1996) Stage-specific accumulation of the terminal differentiation factor LIN-29 during *Caenorhabditis elegans* development. *Development* 122(8):2517-2527.

14. Raj A, van den Bogaard P, Rifkin SA, van Oudenaarden A, & Tyagi S (2008) Imaging individual mRNA molecules using multiple singly labeled probes. *Nat Methods* 5(10):877-879.

15. Boxem M, Srinivasan D, & Van den Heuvel S (1999) The *Caenorhabditis elegans* gene *ncc-1* encodes a cdc2-related kinase required for M phase in meiotic and mitotic cell divisions, but not for S phase. *Development* 126(10):2227-2239.
